# The Earliest Post-Paleozoic Freshwater Bivalves Preserved in Coprolites from the Karoo Basin, South Africa

**DOI:** 10.1371/journal.pone.0030228

**Published:** 2012-02-02

**Authors:** Adam M. Yates, Frank H. Neumann, P. John Hancox

**Affiliations:** 1 Bernard Price Institute for Palaeontological Research, University of the Witwatersrand, Johannesburg, South Africa; 2 Museum of Central Australia, Araluen Cultural Precinct, Alice Springs, Northern Territory, Australia; 3 Forschungsstelle für Paläobotanik, Westfälische Wilhelms-Universität, Münster, Germany; Raymond M. Alf Museum of Paleontology, United States of America

## Abstract

**Background:**

Several clades of bivalve molluscs have invaded freshwaters at various times throughout Phanerozoic history. The most successful freshwater clade in the modern world is the Unionoida. Unionoids arose in the Triassic Period, sometime after the major extinction event at the End-Permian boundary and are now widely distributed across all continents except Antarctica. Until now, no freshwater bivalves of any kind were known to exist in the Early Triassic.

**Principal Findings:**

Here we report on a faunule of two small freshwater bivalve species preserved in vertebrate coprolites from the Olenekian (Lower Triassic) of the Burgersdorp Formation of the Karoo Basin, South Africa. Positive identification of these bivalves is not possible due to the limited material. Nevertheless they do show similarities with Unionoida although they fall below the size range of extant unionoids. Phylogenetic analysis is not possible with such limited material and consequently the assignment remains somewhat speculative.

**Conclusions:**

Bivalve molluscs re-invaded freshwaters soon after the End-Permian extinction event, during the earliest part of the recovery phase during the Olenekian Stage of the Early Triassic. If the specimens do represent unionoids then these Early Triassic examples may be an example of the Lilliput effect. Since the oldest incontrovertible freshwater unionoids are also from sub-Saharan Africa, it is possible that this subcontinent hosted the initial freshwater radiation of the Unionoida. This find also demonstrates the importance of coprolites as microenvironments of exceptional preservation that contain fossils of organisms that would otherwise have left no trace.

## Introduction

The End-Permian extinction event at the end of the Permian Paleozoic Era is widely acknowledged to be the most severe biotic crisis to hit the Earth during the entire Phanerozoic Eon [1]. One of the characteristics of this event is that although its onset was sudden [2], [3], the environmental conditions remained harsh for several million years into the succeeding Triassic Period. These harsh conditions are reflected by the depauperate nature of Early Triassic ecosystems [1], [4], [5] and the small sizes of their constituent species [6]–[8], a phenomenon known as the Lilliput effect [9]. As a consequence there are several gaps in the fossil record of the Early Triassic that attest to the absence of several niches during this inimical stage. The absence of coals and corals from the Early Triassic are well known major examples [10], [11]. Less well studied is the apparent absence of freshwater bivalves during the Early Triassic. Indeed the earliest freshwater bivalves of the Triassic are usually cited to be those from the Late Triassic of the Chinle Formation in North America (e.g. [12], [13]), which are at least 35 million years younger than the extinction event based on recent radiometric dates from the formation [14]. However there are reported, but largely overlooked, occurrences of freshwater unionoids in the Middle Triassic (Anisian) Manda and Ntawere Formations of Tanzania and Zambia, respectively [15], [16]. These are probably no more than 10 million years younger than the extinction event.

Numerous clades of bivalve mollusc have invaded freshwater habitats through Phanerozoic time [17], although none has been as successful in this realm as the Unionoida. We use the taxon Unionoida to denote the stem-based clade that is the sister group to the marine Trigonoida. The close relationship between Unionoida and Trigonoida (as Palaeoheterodonta) among extant bivalves is well supported by phylogenetic analysis of DNA sequence data [18] and combined analyses of DNA and morphology [19]. The unionoids were part of the bivalve re-invasion of freshwaters after the End-Permian extinction event had wiped out earlier freshwater bivalve families. The clade has remained a major part of freshwater habitats throughout the rest of the Mesozoic and Cenozoic Eras. There are approximately 840 extant species of unionoid spread across all continents except Antarctica [20]. Although some Carboniferous and Permian freshwater families have been placed in Unionoida this seems to have been a matter of classification convenience rather than a reflection of a strong phylogenetic hypothesis. Indeed these Paleozoic families appear to have lacked an internal nacreous layer [21] or, given their sporadic occurrences, an elaborate unionoid-like larval dispersal stage [22]. While the lack of a larval stage specialized for dispersal may simply be primitive, the lack of nacre is unexpected in early unionoids given that the Trigonoida are also nacreous. This indicates that these Carboniferous and Permian freshwater bivalves are probably not ancestors of the Triassic unionoids. Indeed it is probably the marine schizodian-grade, or myophorian-grade, palaeoheterodonts of the Permian that were the immediate ancestors of both extant trigonoids and unionoids [23], although this has yet to be tested in a comprehensive cladistic analysis.

Many questions still remain about the early origins of Unionoida and its invasion of the freshwater realm. Included are such basic questions as which fossil palaeoheterodonts, if any, are stem-group unionoids, how soon after the End-Permian extinction event did the unionoids first spread into freshwater, and what was the morphology of the earliest freshwater unionoids?

**Figure 1:**
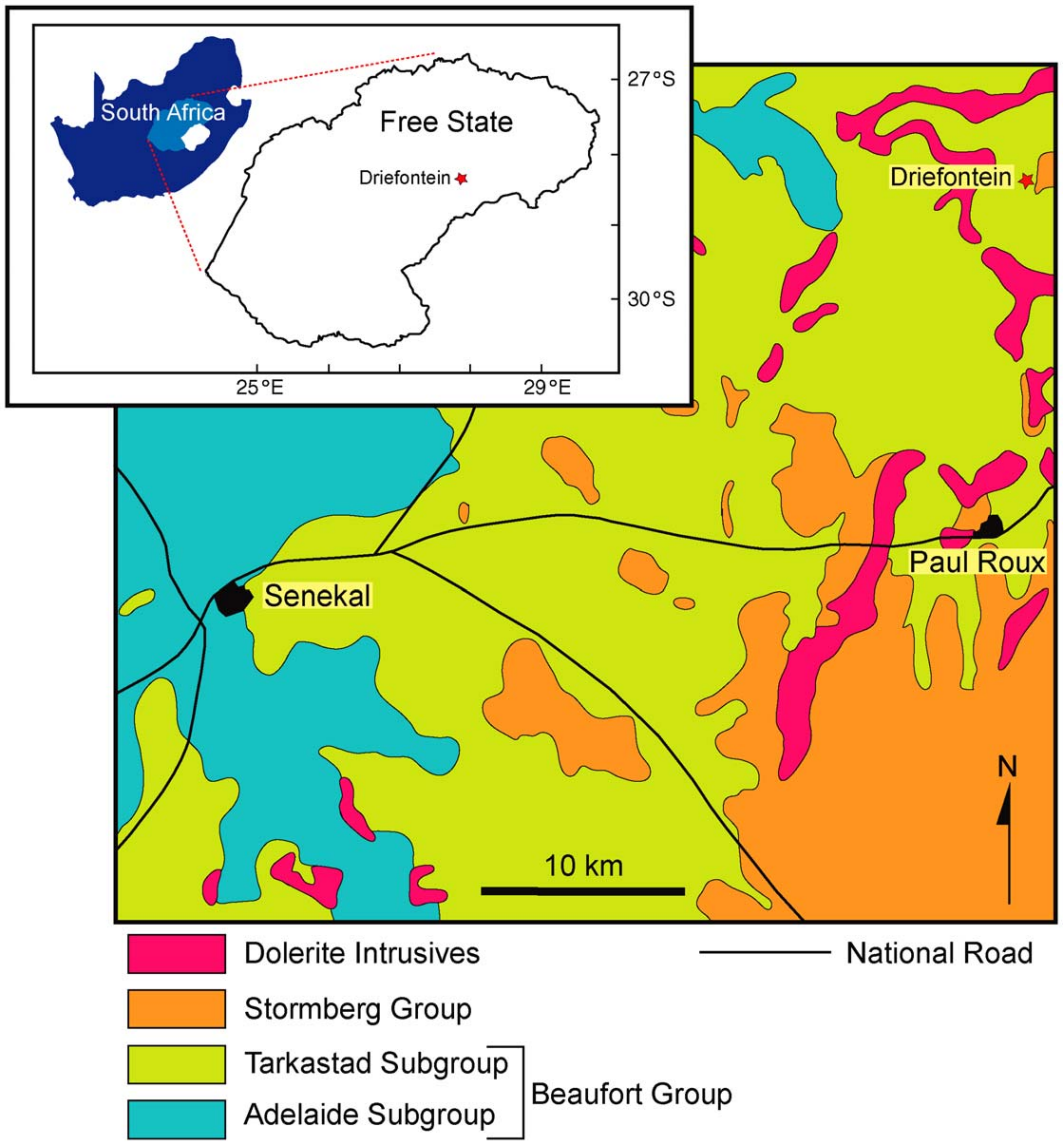
Simplified geological map of the Senekal and Paul Roux districts of the Free State, South Africa showing the location of the Driefontein site. Inset shows the location of the area within the South Africa.

Here we document a remarkable faunule of freshwater bivalves preserved in coprolites from the Early Triassic lower Burgersdorp Formation of the Karoo Basin, South Africa (Figure 1) that may help answer the latter two questions. These are the earliest known post-Paleozoic freshwater bivalves. They may also represent the earliest known freshwater unionoids although this identification must remain rather speculative given the poor quality of the material at hand.

### Geological Setting

The Burgersdorp Formation is a fluvio-lacustrine sequence of siliciclastic rocks at the top of the Tarkastad Subgroup (Beaufort Group) that were deposited in the main Karoo Basin from the Early Triassic through to the early Middle Triassic [24]–[26]. In terms of vertebrate biozonation the formation encompasses the *Cynognathus* Range Zone [27], although there are three temporally distinct vertebrate assemblages within this zone that have been informally labeled subzones A, B and C [28]. The lowermost of these subzones, subzone A, has also been called the ‘*Kestrosaurus* assemblage zone’ [29], although no such biozone has been officially accepted by the South African Committee for Stratigraphy (SACS). The specimens described in this paper were collected at a site on the farm Driefontein 11 in the Nketoana Local Municipality of the eastern Free State (Fig. 1). The presence of the temnospondyls *Parotosuchus haughtoni* [30], *Trematosuchus sobeyi* [31] and the cynodont *Langbergia modisei* [32] at Driefontein or at the same level on a neighboring farm, coupled with the absence of *Xenotosuchus africanus*, *Kannemeyeria simocephalus* and *Erythrosuchus africanus* indicate that the Driefontein locality can be assigned to the ‘*Kestrosaurus* assemblage zone’ [26]. The ‘*Kestrosaurus* assemblage zone’ correlates with the Olenekian stage of the Early Triassic on the basis of vertebrate biostratigraphy [26].

**Figure 2:**
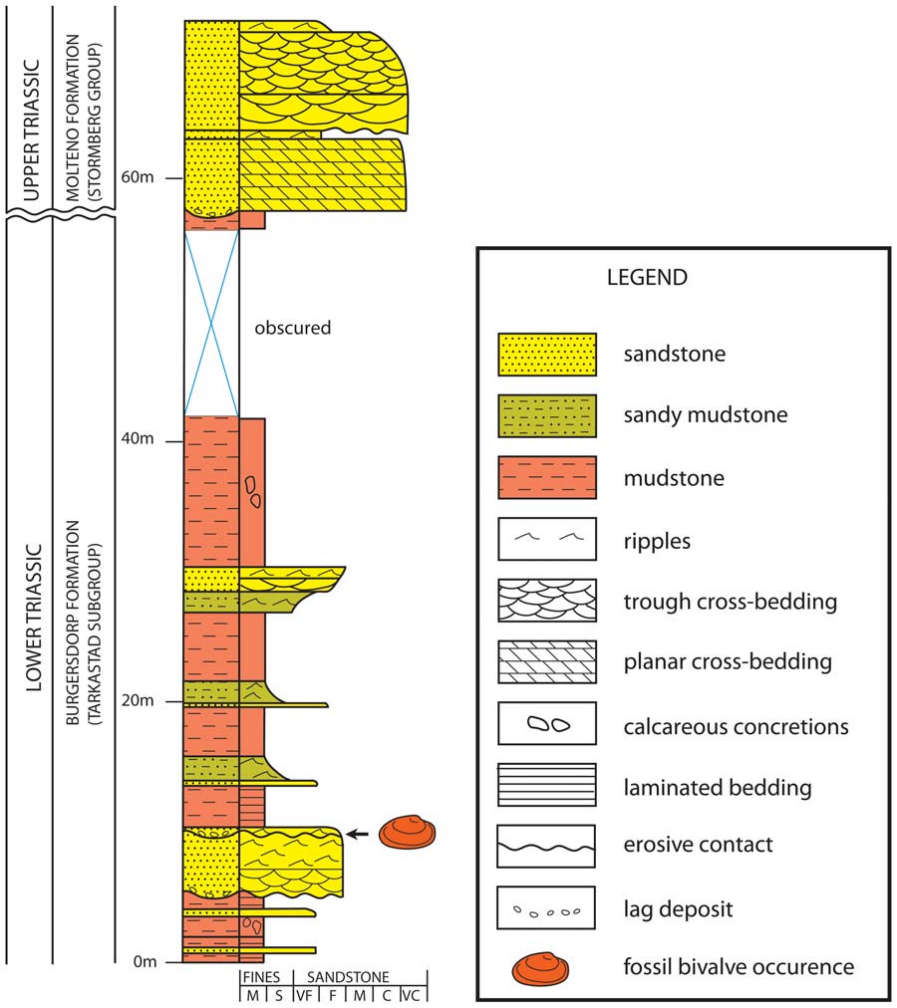
Stratigraphic column of the Driefontein site, showing the position of the bivalve fossils.

**Figure 3:**
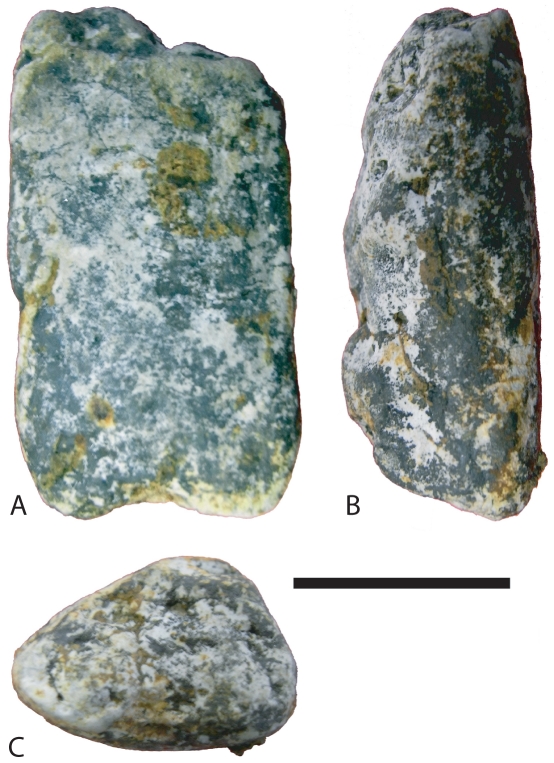
Coprolite from Driefontein. This coprolite shows the subcylindrical shape and flattened base that typical of the type of coprolite that bore BP/1/7047. Scale bar equals 20 mm.

The Driefontein locality incorporates finely laminated lacustrine muds at its base, overlain by a package of channelized sandstones with prominent lag layers developed at the base of several of the channels (Figure 2). These channels indicate that the lake had dried up; perhaps due to a drop in base-level and that the upper-most lacustrine sediments had been scoured and redeposited creating the fossil rich lags. The bivalve specimens described in this paper come from a highly fossiliferous lag that reaches 30 cm in thickness (Figure 2). This particular lag contains abundant fish remains [33], indicating that the assemblage is largely derived from an aquatic fauna. In addition to the vertebrate remains the lag also contains numerous vertebrate coprolites (Figure 3), including spiral forms produced by fish [33].

## Materials and Methods

The stratigraphic section at Driefontein was measured using a Jacob staff and standard geological field techniques. A sample of more than 6000 coprolites was collected from the fossiliferous lag. The surfaces of all coprolites with a diameter greater than 5 mm were examined for exposed organic inclusions using a stereoscopic light microscope. Coprolites with included bivalve remains were mounted on stubs using Dag 580 electroconductive adhesive paint. The stubs were examined and measured using an FEI Quanta 400 E SEM. Acid dissolution and thin sectioning of selected coprolites was also carried out but these did not yield any further information relating to the bivalves.

## Results and Discussion

### Mode of Preservation

Three bivalve-bearing coprolites were found, each with a single bivalve specimen. The most complete specimen including left and right valves was preserved in a large subcylindrical coprolite with a flattened oval cross-section. The coprolite has a transverse diameter of 24 mm, placing it amongst the largest coprolites present at Driefontein. The coprolite producer is unknown but a large saurichthyid fish, temnospondyl amphibian, or basal archosauriform reptile are plausible producers. The other two specimens are irregular, roughly equant rounded masses of coprolitic material with a maximum diameter between 10 and 15 mm, each with a single fragment of bivalve shell. The producers of these coprolites are unknown.

The bivalve specimens were preserved as moulds inside vertebrate coprolites from the thick, uppermost lag layer. It is important to note that the coprolites provided a medium capable of preserving the thin shells of these molluscs whereas the surrounding siliclastic sediments contain no trace of mollusc shells. It is possible that these were lost during early diagenetic leaching and it was only those that were protected by already mineralised coprolites that have survived. This serves to highlight the importance of coprolites as microenvironments of exceptional preservation.

### Descriptions

#### BP (Bernard Price Institute for Palaeontological Research, University of the Witwatersrand, Johannesburg, South Africa)/1/7047 (Figures 4, 5, 6)

**Figure 4:**
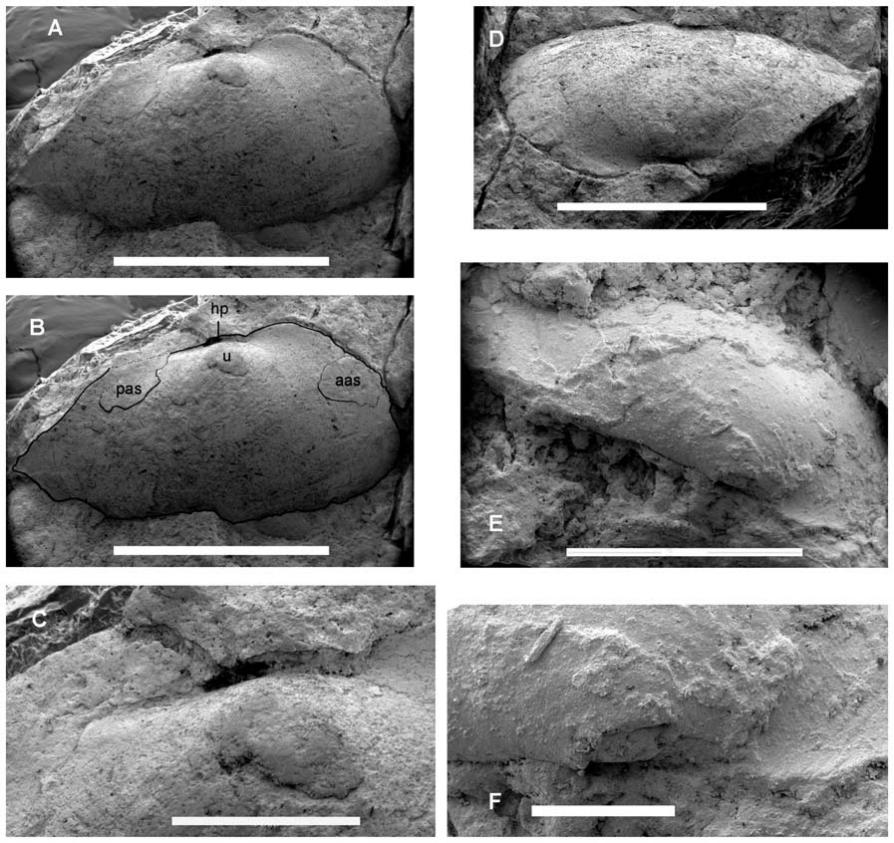
SEM images of Bivalvia indet. cf. Unionoida (BP/1/7047). **A.** Internal mould of right valve in lateral view. **B.** Interpretation of the preserved features of the right valve. **C.** Close-up of the umbonal region of the right valve. **D.** Dorsolateral view of the internal mould of the right valve. **E.** Silcone rubber cast of the external mould of the left valve in dorsal view. **F.** Close-up of the umbonal region of the left valve. **Abbreviations: aas**, anterior adductor scar; **hp**, hinge plate; **pas**, posterior adductor scar; **u**, umbo. Scale bars in **A**, **B**, **D** and **E** equal 4 mm, those in **C** and **F** equal 1 mm.

**Figure 5:**
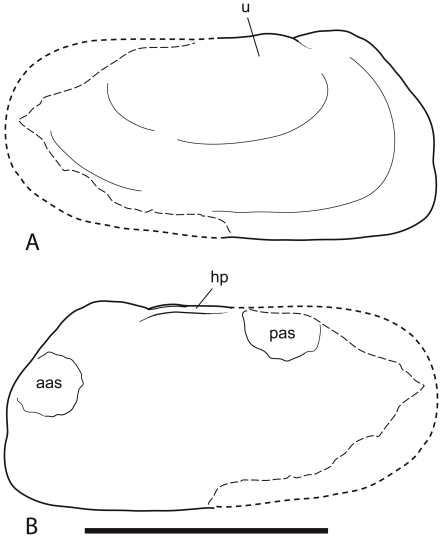
Reconstruction of the right valve of Bivalvia indet. cf. Unionoida (BP/1/7047). **A.** External view. **B.** Internal view. **Abbreviations: aas**, anterior adductor scar; **hp**, hinge plate; **pas**, posterior adductor scar; **u**, umbo. Scale bar equals 4 mm.

**Figure 6:**
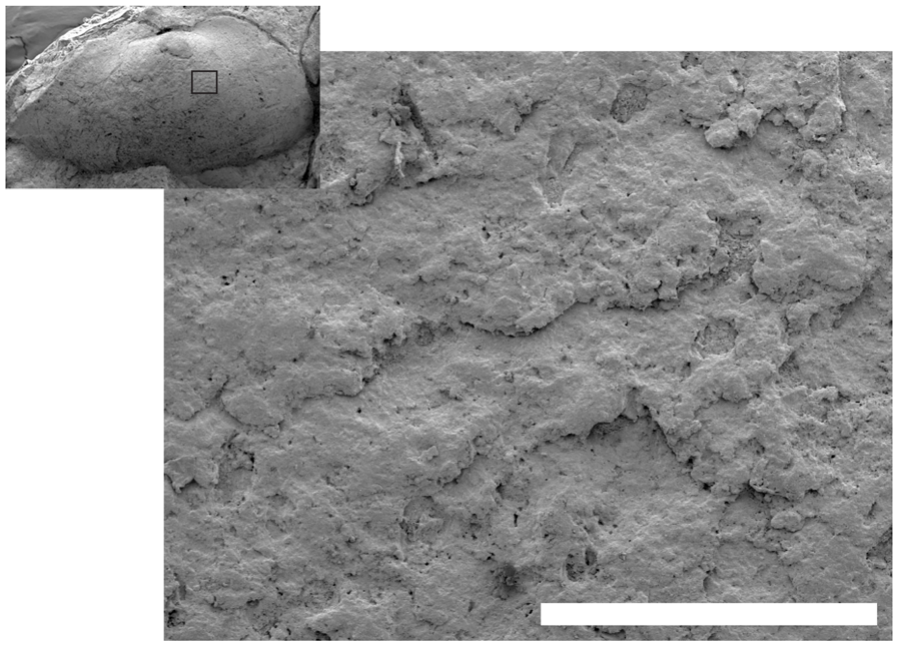
Possible ultrastructural impressions of nacre on (BP/1/7047). Close up of the anterior subumbonal region of the internal mould of the right valve showing poorly-preserved sheet-like structures. Inset shows the position of the close-up. Scale bar equals 200 µm.

The coprolite contains an internal mould of the right valve and an external mould of the left valve. The dimyarian shell appears to have been close to equivalve and moderately inflated. Each valve is transversely elliptical and approximately twice as long as it is high. The anterior end is distinctly truncate while the posterior end is produced well beyond the hinge plate and the posterior adductor muscle scar. The posterior end of the valve is evenly convex and does not include a distinct posterior ridge separating a posterior area. The umbos are placed approximately one third of the length of the shell from the anterior end and barely protrude above the dorsal margin of the hinge plate. The lunular area is developed into dorsally projecting flange that is apparently larger in the right valve than in the left. The external mould of the left valve indicates that there was no sculpturally differentiated lunule. The hinge plate itself is both narrow and short. Poor preservation obscures details of the hinge such that it cannot even be determined if it was edentulous or not. Nevertheless it is clear that any hinge teeth present would have had to have been rather weak given the narrowness of the hinge plate. A posterior adductor muscle scar lying against the dorsal margin can be seen against the dorsal margin of the right valve. An extremely faint anterior muscle scar appears low down on the anterior margin in a position that is quite distant from the anterior end of the hinge plate. Externally the shell is smooth but for a few incomplete, weakly developed comarginal growth lines. There are small patches of scarp-like ridges preserved in the umbonal area of the right valve (Figure 6). These are spaced approximately 60 to 110 µm apart and are approximately 2–5 µm high. The size of these is consistent with the impressions of the individual sheets of crystallites that comprise sheet nacre [34], but their poor preservation and sporadic occurrence leaves such an interpretation inconclusive.

#### BP/1/7048 (Figure 7)

**Figure 7:**
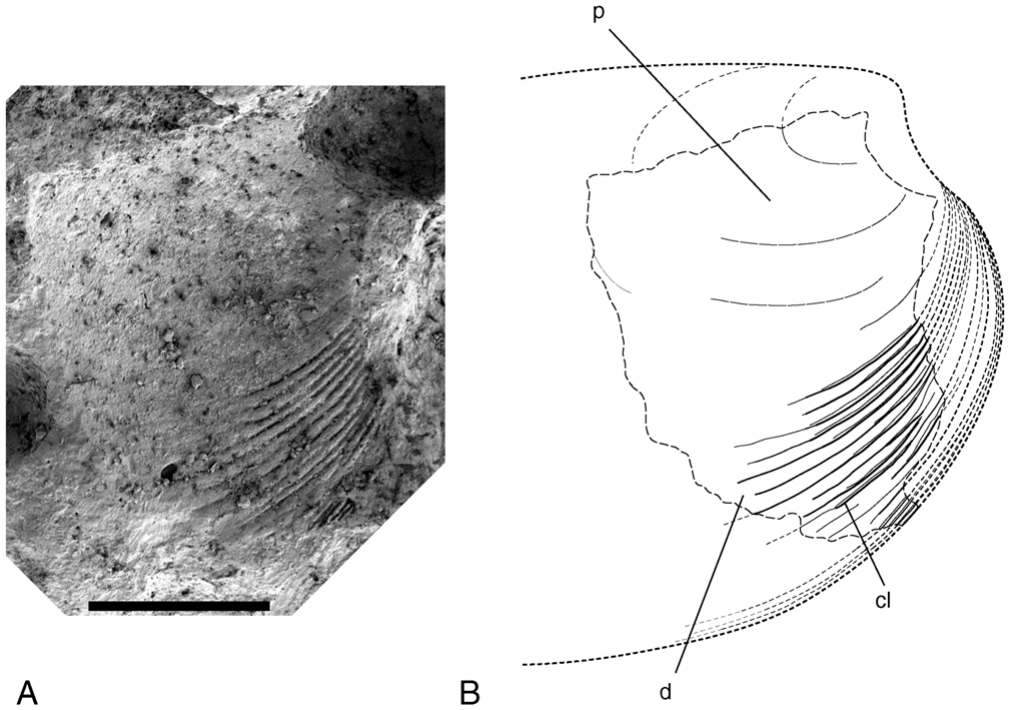
External mould of a fragment of Bivalvia indet. cf. Unionoida (BP/1/7048). **A.** SEM image. **B.** Reconstruction. Scale bar equals 1 mm.

The coprolite bears a single, incomplete external mould of a left valve. It is clear from the external ornament that it does not belong to the same species as BP/1/7047. Little of the shape can be made out but it would appear that the beak was more protrusive than in BP/1/7047. The external ornament is abruptly divided into two regions of differing external ornament, here taken to represent the prodissoconch and the dissoconch. The prodissoconch is smooth and crossed only by weak comarginal growth lines. It reaches a maximum height of 1.66 mm as preserved. Because only the very tip of the beak is missing, this dimension is likely to be very close to the maximum height of the larval shell before the onset of mature shell growth. The anterior end of the dissoconch is strongly sculpted with thin, closely spaced, concentric lamellae. The lamellae were just 15 microns thick and reach a maximum spacing of 35–62 microns apart. The three lamellae adjacent to the margin of the shell are more closely spaced than the others, indicating that growth had slowed and adult size had been reached. The lamellae fade rapidly in prominence towards the middle of the disc and are reduced to simple comarginal growth lines at the posterior end of the shell fragment.

#### BP/1/7049

Little can be said of this specimen. It is a small fragment of shell represented only as an impression of the external surface. Like BP/1/7047 it is essentially smooth and crossed only by weak comarginal growth lines and may belong to the same species as that specimen.

### Systematic Position of the Driefontein Bivalves

**Figure 8:**
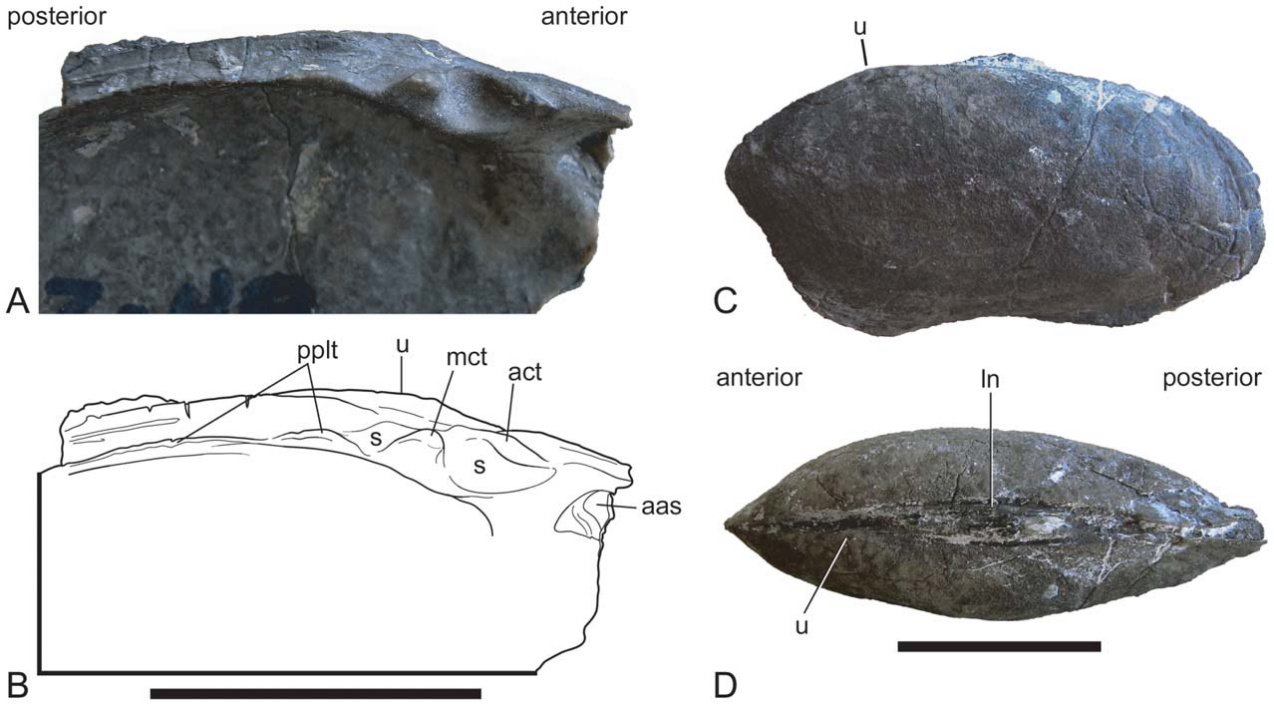
‘*Unio*’ *karooensis*, Manda Formation, Tanzania. **A.** Hinge plate of an isolated left valve (FMNH Z.48). **B.** Interpretative drawing of A. **C.** Conjoined pair of valves (FMNH Z.31) in left lateral view. **D.** FMNH Z.31 in dorsal view. **Abbreviations: aas**, anterior adductor scar; **act**, anterior cardinal tooth; **e**, escutcheon; **ln**, ligamental nymph; **mct**, median cardinal tooth; **pplt**, posterior pseudolateral tooth; **s**, socket; **u**, umbo. Scale bar equals 20 mm.

The low numbers of specimens obtained and their generally poor state of preservation makes a firm identification impossible. However, we believe it is probable that these fossils belong to the freshwater clade of the Unionoida for the following reasons. Firstly the transversely elongate elliptical shell shape of BP/1/7047 (Figure 5) matches the typical shape of unionoids, including ‘*Unio*’ *karooensis* from the Middle Triassic of Tanzania. We believe ‘*Unio*’ *karooensis* to be a true member of the freshwater unionoid clade based on its fluviatile provenance, elongate ligament and shizodont hinge with an elongate posterior pseudolateral tooth (Figure 8). Although details of the hinge of the South African fossils cannot be seen, it is clear that the dentition was poorly developed, if it was present at all, a characteristic seen in several crown-group unionoids. The strong distinction between the sculpture of the prodissoconch and the dissoconch of BP/1/7048 is also reminiscent of many crown-group unionoids where the sculptural change represents the change from the parasitic larval stage to the final benthic stage, although it must be noted that in extant unionoids it is the prodissoconch that is more strongly sculptured. As discussed above, there are some indications that the inner shell layer was nacreous, another feature in common with unionoids, albeit a primitive retention. Lastly we find the proximity of these bivalves in time and space to the earliest incontrovertible freshwater unionoids highly suggestive. Whether or not these fossils are freshwater unionoids, their early age and small size make it unlikely that they are members of crown-group unionoids. None of our observations are conclusive and our inability to code most shell-based characters, including those of the hinge teeth, would make any attempt at phylogenetic analysis futile. It is only through better quality fossil bivalves from the ‘*Kestrosaurus* assemblage zone’ that we will be able to make a firmer identification.

Nonetheless, if a unionoid identification for these bivalves can be upheld, a number of significant implications are raised. Firstly they would be the oldest known freshwater members of the group and would indicate that the clade invaded freshwater earlier than previously suspected, right in the biotic recovery phase from the end-Permian extinction event. They would also suggest that central Gondwana may have hosted this initial invasion. Their small size may also indicate that the earliest freshwater unionoids may have undergone a reduction in size, the so-called ‘Lilliput effect’ observed in other Early Triassic molluscs. Each of these ideas is discussed in more detail below.

### Freshwater Bivalves During the Triassic Recovery Phase

The Driefontein bivalves demonstrate that the freshwater bivalve niche was occupied during the recovery phase of the end-Permian extinction event, at least in sub-Saharan Africa. The presence of two distinct taxa further suggests that a modest amount of cladogenesis had occurred and that bivalves, probably unionoids, had invaded freshwaters no later than the early Olenekian; at the very beginning of the biotic recovery. However conditions in this niche may have been harsh and consequently the only known specimens are unusually small (less than a centimeter in length). By the Anisian Stage, at the end of the recovery phase, unionoid bivalves were both large and abundant in fluviatile faunas of Africa. The shells of ‘*Unio*’ *karooensis* are large and robust and have a high preservation potential so it is unusual that similar unionoids have not been recovered from other continental Middle Triassic deposits. It may be that at this early stage of their evolution freshwater unionoids were restricted to central Gondwana. It is important to note that the invasion and establishment of freshwater unionoids in Africa that is suggested here need not be coincident with the radiation of crown-group unionoids and thus has no impact on current hypotheses relating to the biogeography of extant unionoid families.

### Size of the Driefontein Bivalves

The adult size range of extant unionoids extends from 25 mm to 300 mm [35], and thus exceeds the size of the Driefontein bivalves (Table 1). The closely packed concentric ornament at the margin of BP/1/7048 suggests adulthood had been reached which indicates that, at least for this species, the small size of the Driefontein bivalves is not due simply to a preservational bias toward juvenile shells. ‘*Unio*’ *karrooensis*, the earliest definite freshwater unionoid, from the middle Triassic, has a typical size for extant unionoids (55–93 mm; pers. obs. of FMNH (Field Museum, Chicago, USA) Z-31, Z-38). Size reduction of individuals after biotic crises is a recognized phenomenon, and the term ‘Lilliput effect’ has been coined for it [9]. The effect likely had multiple variable causes with low concentrations of atmospheric oxygen and food shortages, brought on by reduced levels of biological production, being foremost among them [8]. Molluscs seem to have been particularly prone to the Lilliput effect in the wake of the End-Permian extinction event [6]–[8] although some recent discoveries of larger Early Triassic gastropods suggest that the prevalence and/or duration of this effect may not have been as great as initially thought [36]. If the present specimens are accepted as freshwater unionoids, then the larger size of all later freshwater unionoids suggests that the small size of the Driefontein bivalves may also be an example of the Lilliput effect.

**Table 1:** Measurements of the Driefontein bivalves.

*Measurement*	BP/1/7047	BP/1/7048
Length (as preserved)	7.05 mm	2.36 mm
Estimated total length	7.30 mm	-
Height (as preserved)	3.54 mm	2.42 mm
Estimated total height	3.60 mm	
Hinge length	∼1.5 mm	-
Distance from umbo to anterior end	2.82 mm	-
Shell thickness	65.6–77.9 µm	-

Shell thickness is measured as the distance between the internal and external mould at the posterior end of the right valve of BP/1/7047.

### Paleoecological Considerations

The bivalve-bearing lag appears to contain reworked specimens from the underlying lacustrine muds but may well contain the fossils of some channel-dwelling fauna as well. However, it is probable that the coprolites themselves were permineralized in dysaerobic lacustrine muds, below the sediment-water interface. This is an inference supported by the presence of pyrite pseudomorphs preserved in and around the coprolite specimens. Therefore we can accept the bivalves were part of the lacustrine benthos. The presence of their shells in vertebrate coprolites immediately suggests that these molluscs were a food resource to the coprolite producer. However, not all inclusions found in coprolites necessarily represent food items. Several observations suggest that these occurrences represent incidental or accidental ingestion. Firstly the three coprolites contain a single pair of valves from one individual and a single fragment from one valve respectively. If these coprolites were produced by a molluscivorous predator, then a greater density of shells within the fecal mass would be expected. Secondly the valves of BP/1/7047 are largely complete. A finer trituration of the shell would be expected from a predator intent on feeding upon the bivalve. A possible scenario was that the bivalves were accidentally ingested when some of the lake sediment was scooped up during a predatory lunge made by one of the many larger piscivorous vertebrate taxa known from the site (e.g. saurichthyid fish, basal archosauriforms, mastodonsauroid and brachyopid temnospondyls). This scenario would be more plausible if these bivalves were abundant faunal elements of the benthos, thus increasing the chance of accidental ingestion. Thus there is weak evidence that freshwater bivalves were common in the Driefontein lake.
